# Supplementary material for: Medium-to-Long-Term Immunogenicity of BNT162b2 mRNA COVID-19 Vaccine: A Retrospective Cohort Study
Source: Vaccines (Basel). 2022 Mar 10;10(3):417. doi: 10.3390/vaccines10030417 (PMC8949567; doi:10.3390/vaccines10030417)
Supplement: Supplementary file 1 [file vaccines-10-00417-s001.zip › vaccines-1605542-supplementary.pdf]

Figure S1. Trend in the GMT (95%CI) of anti-COVID19 IgG in screened HCWs, per sex.

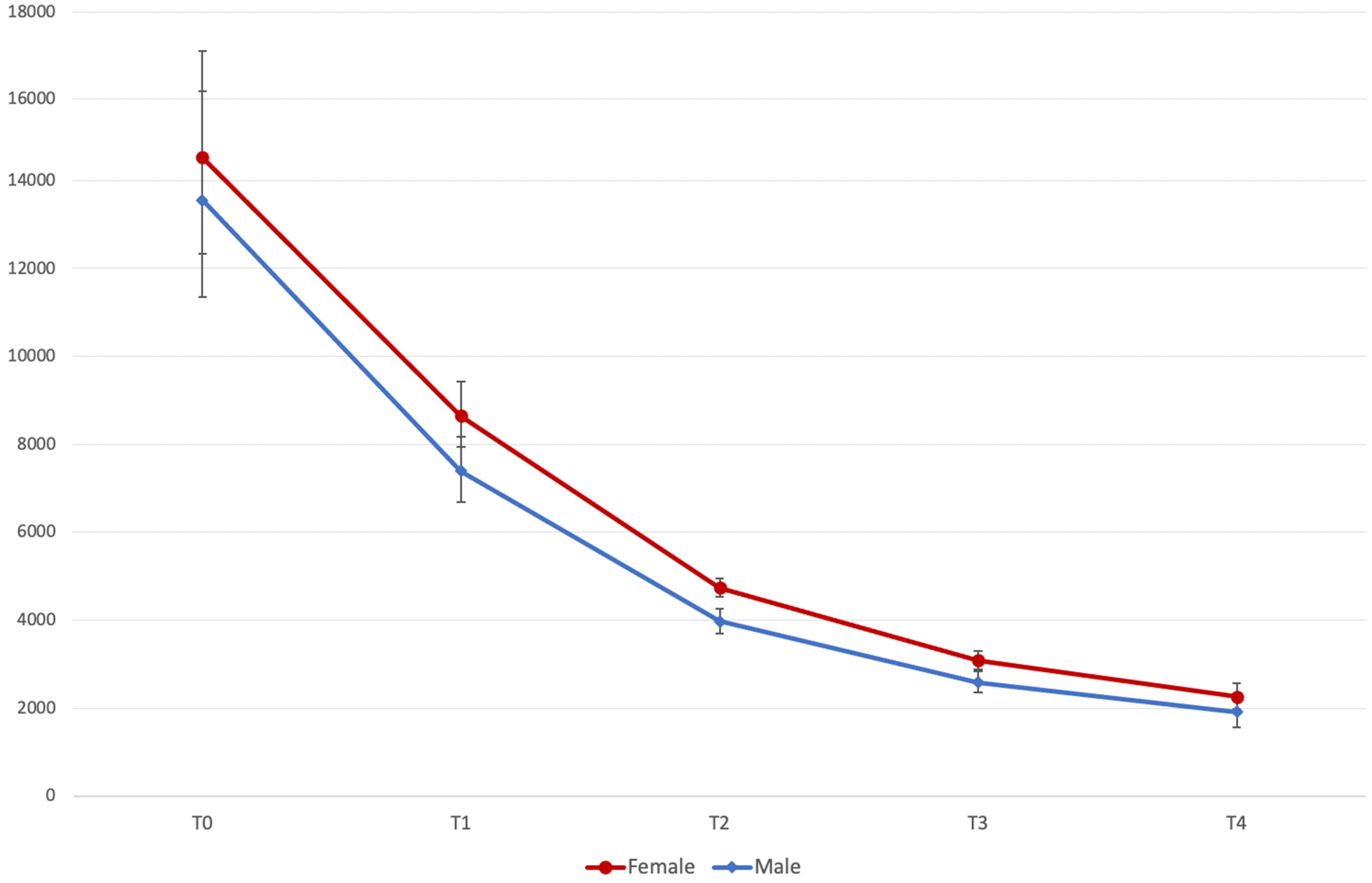

Figure S2. Trend in the GMT (95%CI) of anti-COVID19 IgG in screened HCWs, per age class.

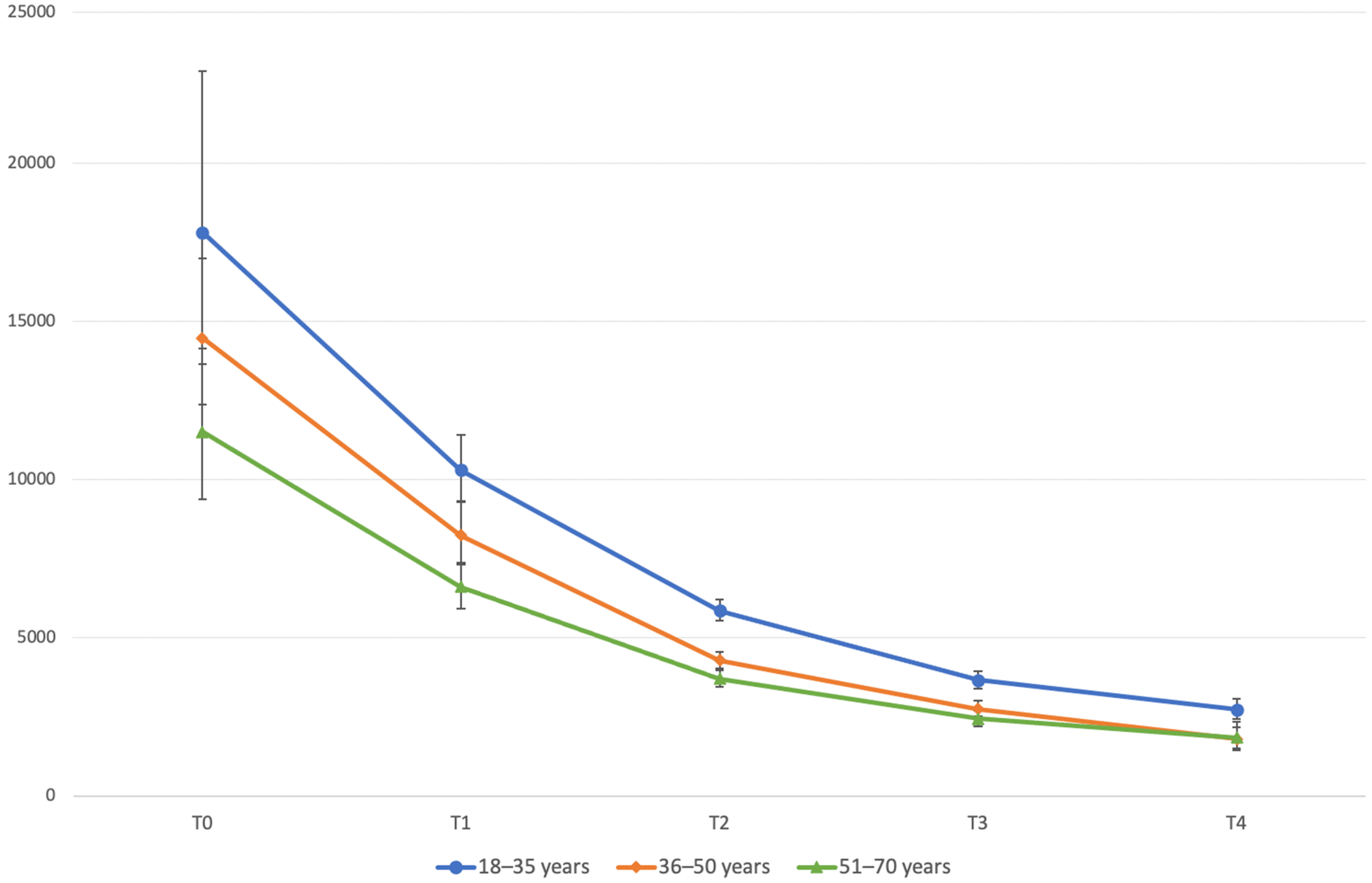

Figure S3. Trend in the GMT (95%CI) of anti-COVID19 IgG in screened HCWs, per job type.

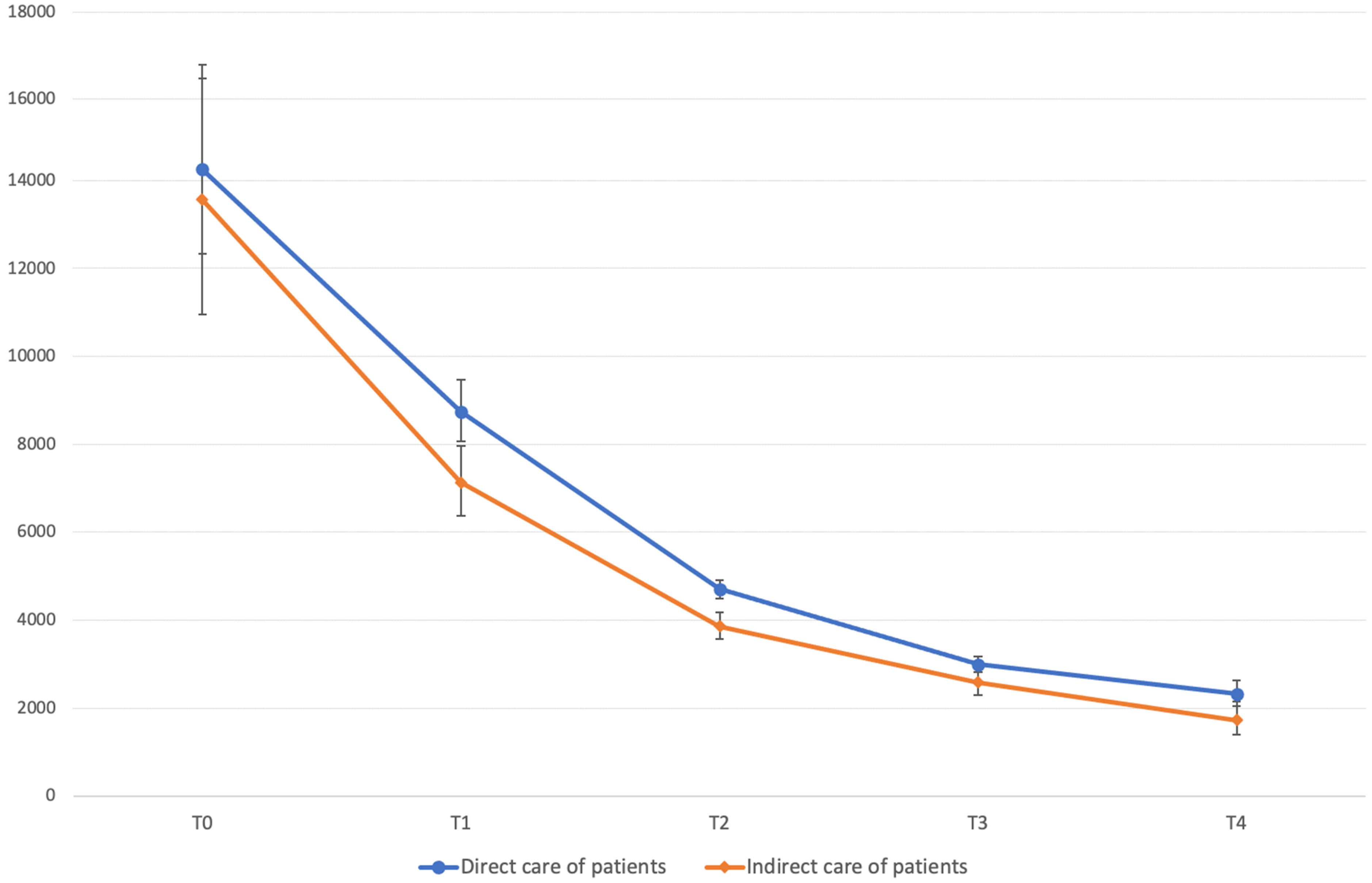

Figure S4. Trend in the GMT (95%CI) of anti-COVID19 IgG in screened HCWs, per ward.

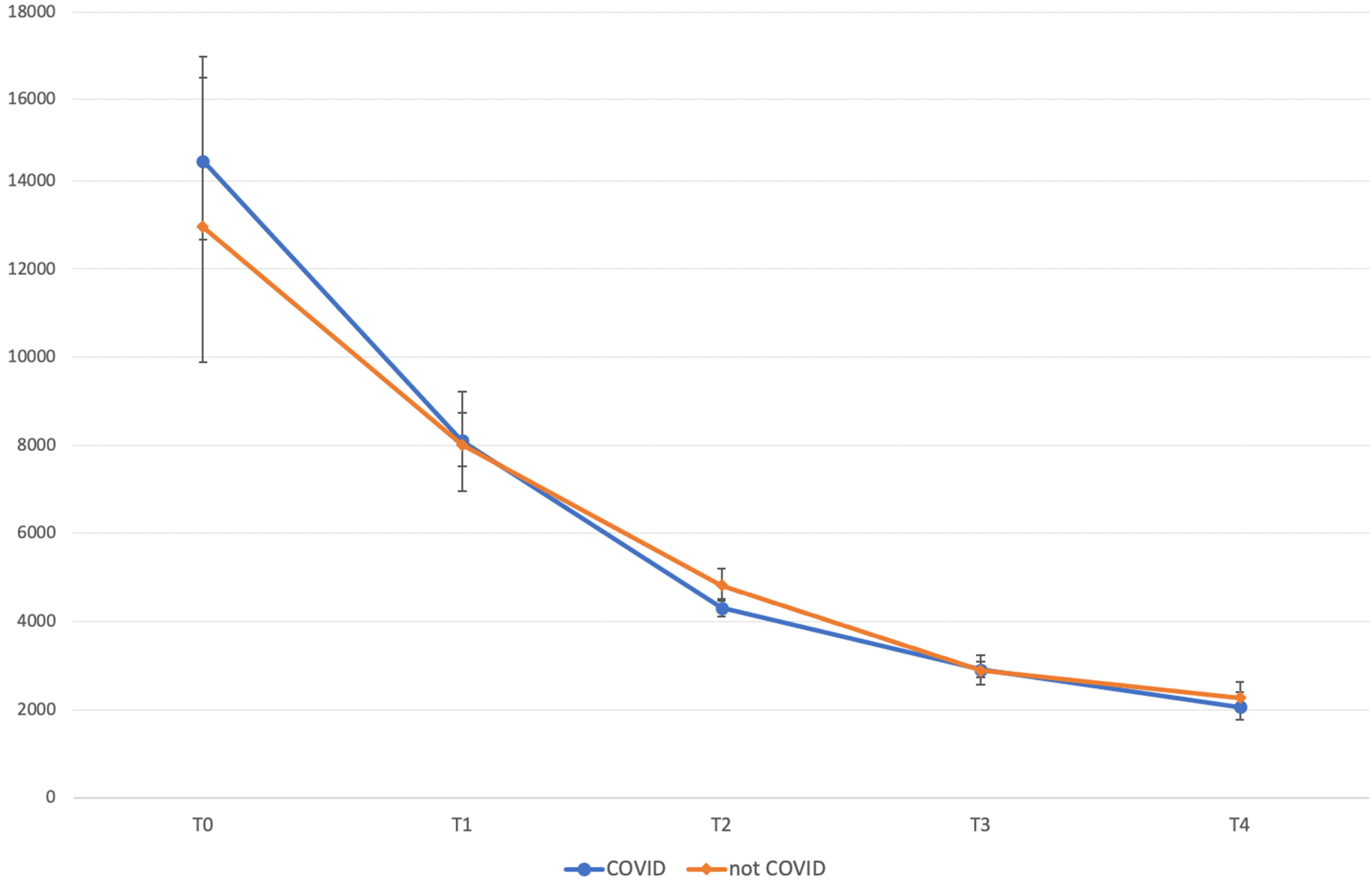

Table S1. Tests of simple effects to explain the significant interaction between sex and time.

	Contrast	95% confidence interval	Standard error	p-value
**Group**				
Male vs. female	−620.6	−1,174.4 – −66.8	282.6	0.028
**Time**				
T1 vs T0	−7,218.2	−8,021.0 – −6,413.4	410.6	<0.0001
T2 vs. T0	−12,371.0	−13,105.7 – −11,636.3	374.9	<0.0001
T3 vs. T0	−14,496.7	−15,308.8 – 13,684.7	414.3	<0.0001
T4 vs. T0	485.1	−16,466.6 – −14,564.9	485.1	<0.0001
**Group×time**				
(male vs. female)×(T1 vs. T0)	−1,659.4	−3,268.9 – −49.8	821.2	0.043
(male vs. female)×(T2 vs. T0)	−587.8	−2,057.3 – 881.7	749.7	0.433
(male vs. female)×(T3 vs. T0)	−333.9	−1,958.0 – 1,290.1	828.6	0.687
(male vs. female)×(T4 vs. T0)	−715.4	−2,617.1 – 1,186.3	970.3	0.461

Table S2. Tests of simple effects to explain the significant interaction between age class and time.

	Contrast	95% confidence interval	Standard error	p-value
**Group**				
36–50 vs. 18–35 yrs	−2252.5	−2,938.6 – −1,566.4	350.1	<0.0001
51–70 vs. 18–35 yrs	−2494.9	−3,132.2 – −1,857.5	325.2	<0.0001
**Time**				
T1 vs. T0	−7,336.5	−8,139.5 – −6,533.6	409.7	<0.0001
T2 vs. T0	−12,574.3	−13,307.6 – −11,841.0	374.1	<0.0001
T3 vs. T0	−14,817.7	−15,617.6 – −14,017.8	408.1	<0.0001
T4 vs. T0	−15,747.5	−16,675.1 – −14,819.9	473.3	<0.0001
**Group×time**				
(36–50 vs. 18–35 yrs)×(T1 vs. T0)	2,264.4	216.2 – 4,312.5	1,045.0	0.030
(36–50 vs. 18–35 yrs)×(T2 vs. T0)	3,185.0	1,321.1 – 5,049.0	951.0	0.001
(36–50 vs. 18–35 yrs)×(T3 vs. T0)	4,076.3	2,045.5 – 6,107.0	1,036.1	<0.0001
(36–50 vs. 18–35 yrs)×(T4 vs. T0)	4,092.1	1,740.8 – 6,443.3	1,199.6	0.001
(51–70vs. 18–35 yrs)×(T1 vs. T0)	3,064.3	1,129.1 – 4,999.6	987.4	0.002
(51–70vs. 18–35 yrs)×(T2 vs. T0)	5,070.2	3,277.1 – 6,6863.4	914.9	<0.0001
(51–70vs. 18–35 yrs)×(T3 vs. T0)	5,697.8	3,756.2 – 7,639.5	990.6	<0.0001
(51–70 vs. 18–35 yrs)×(T4 vs. T0)	6,369.6	4,162.6 – 8,576.5	1,126.0	<0.0001

Table S3. Tests of simple effects to explain the significant interaction between job type (direct care vs. indirect care of patients) and time.

	Contrast	95% confidence interval	Standard error	p-value
**Group**				
Direct vs. indirect	−870.8	−1,463.9 – −277.7	302.6	0.004
**Time**				
T1 vs. T0	−7,178.9	−8,073.8 – −6,284.1	456.6	<0.0001
T2 vs. T0	−12,279.4	−13,111.4 – −11,447.4	424.5	<0.0001
T3 vs. T0	−14,362.8	−15,287.1 – −13,438.4	471.6	<0.0001
T4 vs. T0	−15,413.3	−16,445.1 – −14,381.5	526.4	<0.0001
**Group×time**				
(Direct vs. indirect)×(T1 vs. T0)	−715.9	−2,505.7 – 1,703.9	913.2	0.433
(Direct vs. indirect)×(T2 vs. T0)	280.1	−1,383.9 – 1,944.1	849.0	0.741
(Direct vs. indirect)×(T3 vs. T0)	667.9	−1,180.8 – 2,516.5	943.2	0.479
(Direct vs. indirect)×(T4 vs. T0)	142.9	−1,920.6 – 2,206.6	1,052.9	0.892

Table S4. Tests of simple effects to explain the significant interaction between wards (COVID vs. not COVID) and time.

	Contrast	95% confidence interval	Standard error	p-value
**Group**				
COVID vs. not COVID	−25.0	−645.8 – 595.7	316.7	0.937
**Time**				
T1 vs. T0	−7,059.2	−7,946.4 – −6,172.1	452.6	<0.0001
T2 vs. T0	−12,176.2	−12,978.4 – −11,373.9	409.3	<0.0001
T3 vs. T0	−14,404.6	−15,286.7 – −13,522.6	450.0	<0.0001
T4 vs. T0	−15,400.3	−16,412.4 – −14,388.1	516.4	<0.0001
**Group×time**				
(COVID vs. not COVID)×(T1 vs. T0)	407.8	−1,366.5 – 2,182.2	905.3	0.450
(COVID vs. not COVID)×(T2 vs. T0)	763.2	−841.4 – 2,367.8	818.7	0.930
(COVID vs. not COVID)×(T3 vs. T0)	470.4	−1,293.7 – 2,234.4	900.0	0.520
(COVID vs. not COVID)×(T4 vs. T0)	263.5	−1,760.7 – 2,287.8	1032.8	0.260
